# COVID-19 SeroHub, an online repository of SARS-CoV-2 seroprevalence studies in the United States

**DOI:** 10.1038/s41597-022-01830-4

**Published:** 2022-11-26

**Authors:** Neal D. Freedman, Liliana Brown, Lori M. Newman, Jefferson M. Jones, Tina J. Benoit, Francisco Averhoff, Xiangning Bu, Konuralp Bayrak, Anna Lu, Brent Coffey, Latifa Jackson, Stephen J. Chanock, Anthony R. Kerlavage

**Affiliations:** 1 National Cancer Institute, Rockville, USA; 2 National Institute of Allergy and Infectious Diseases, Rockville, USA; 3 Centers for Disease Control and Prevention, Atlanta, USA

**Keywords:** Viral infection, Epidemiology

## Abstract

Seroprevalence studies provide useful information about the proportion of the population either vaccinated against SARS-CoV-2, previously infected with the virus, or both. Numerous studies have been conducted in the United States, but differ substantially by dates of enrollment, target population, geographic location, age distribution, and assays used. This can make it challenging to identify and synthesize available seroprevalence data by geographic region or to compare infection-induced versus combined infection- and vaccination-induced seroprevalence. To facilitate public access and understanding, the National Institutes of Health and the Centers for Disease Control and Prevention developed the COVID-19 Seroprevalence Studies Hub (COVID-19 SeroHub, https://covid19serohub.nih.gov/), a data repository in which seroprevalence studies are systematically identified, extracted using a standard format, and summarized through an interactive interface. Within COVID-19 SeroHub, users can explore and download data from 178 studies as of September 1, 2022. Tools allow users to filter results and visualize trends over time, geography, population, age, and antigen target. Because COVID-19 remains an ongoing pandemic, we will continue to identify and include future studies.

## Abstract (summary)

**Table:** 

Measurement(s)	SARS-CoV-2 seroprevalence
Technology Type(s)	serology assay
Factor Type(s)	geography • age • sex • race/ethnicity • Collection date
Sample Characteristic - Organism	Homo sapiens
Sample Characteristic - Environment	spatiotemporal region
Sample Characteristic - Location	United States of America

## Background & Summary

Policymakers, researchers, and the general public must be able to track the proportion of the population infected with severe acute respiratory syndrome coronavirus 2 (SARS-CoV-2)^1^ and the proportion vaccinated. Seroprevalence studies that detect the presence of SARS-CoV-2 antibodies in people’s sera^2,3^ provide estimates of the proportion of the population previously infected, vaccinated, or both and may also help to project the proportion of the U.S. population susceptible to the virus.

In response to this need, researchers have conducted numerous SARS-CoV-2 seroprevalence studies. However, such studies vary widely in design. Some national studies used residual clinical laboratory specimens^4,5^ or blood donor specimens^6^ and performed serial measurements over many months. Other studies had a single sample collection period and recruited from specific populations such as health care workers^7^ or residents of specific geographic areas such as states or counties^8–10^. Some studies used convenience sampling^11^, whereas more representative studies used defined sampling frames^9,10^. Likewise, a wide variety of serology assays were used, detecting a range of antibody isotypes and antigen targets (*e.g*., spike or nucleocapsid)^12^. Some studies release their results as part of a single pre-print or peer-reviewed publication. Others^4–6^ release data serially on publicly available websites that can be accessed using Application Programming Interfaces (API).

Several prior efforts have aimed to summarize the SARS-CoV-2 seroprevalence literature, including traditional meta-analysis^13–15^. However, the fast pace of data release in an ongoing pandemic means that real-time updates are needed to complement traditional meta-analyses. One effort, Serotracker.com^16,17^ tracks seroprevalence studies conducted around the world and presents results in an online dashboard. However, these efforts do not allow users to easily visualize how seroprevalence varies by antigen target, calendar time, or geography. Within the United States, infection and vaccination rates substantially differ by geographic region, and it is important to allow users to examine these differences by displaying results at state and local levels.

**Fig. 1:**
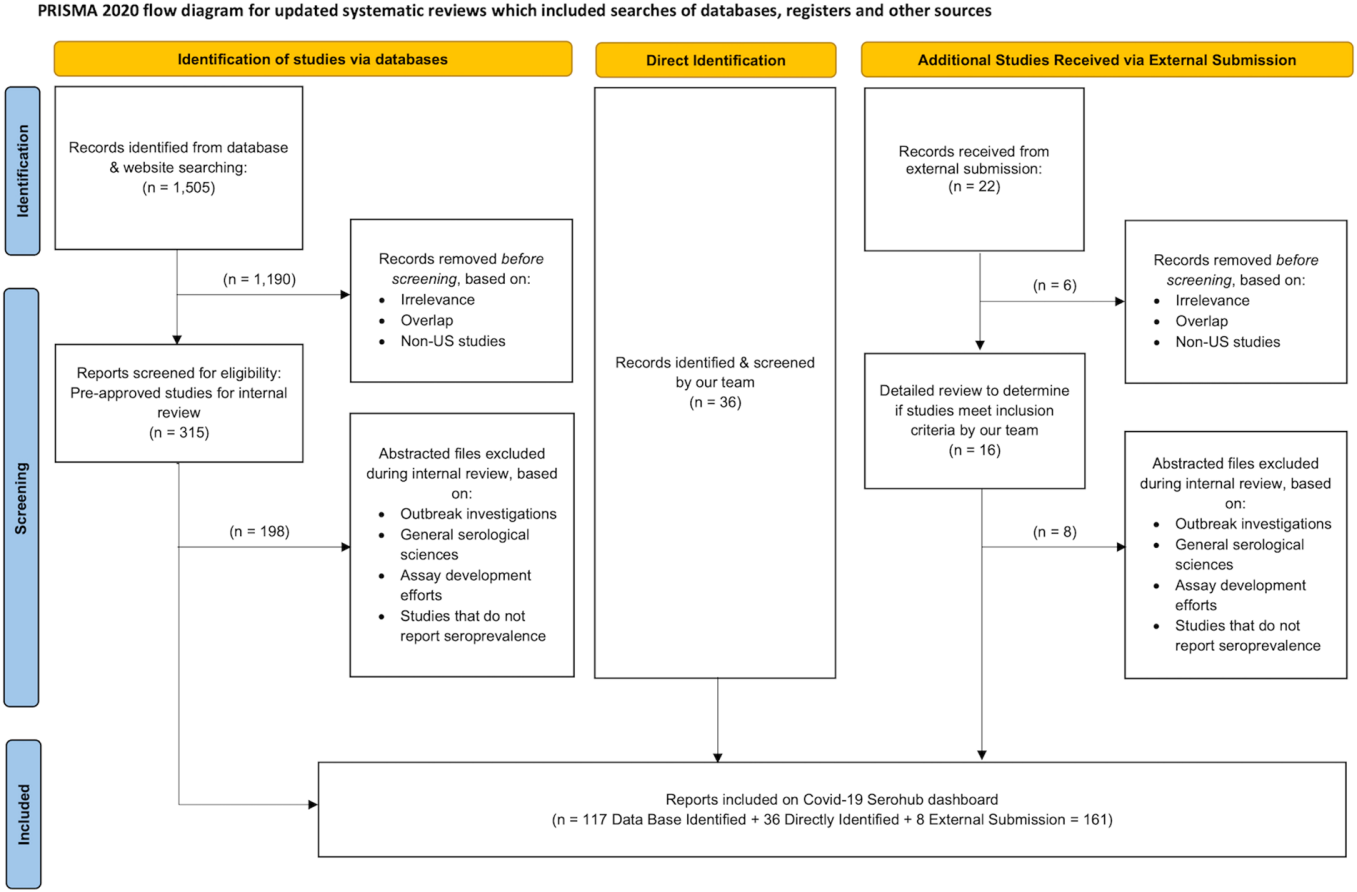
Flow chart indicating how studies were identified for inclusion in COVID-19 SeroHub.

**Fig. 2:**
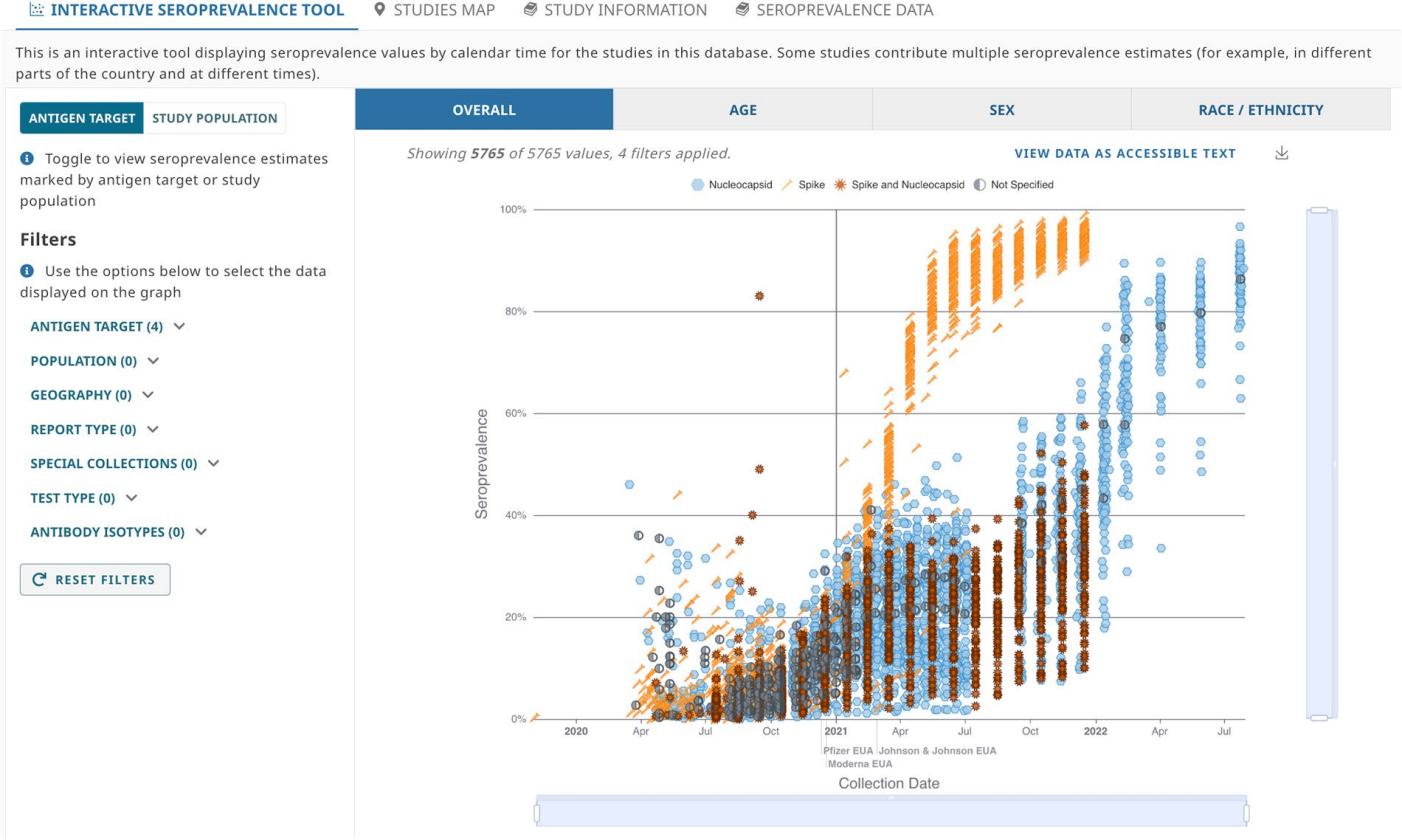
Screenshot of the Interactive Seroprevalence Tool in COVID-19 SeroHub that allows users to view seroprevalence results by calendar time, geography, antigen target, and other factors.

**Fig. 3:**
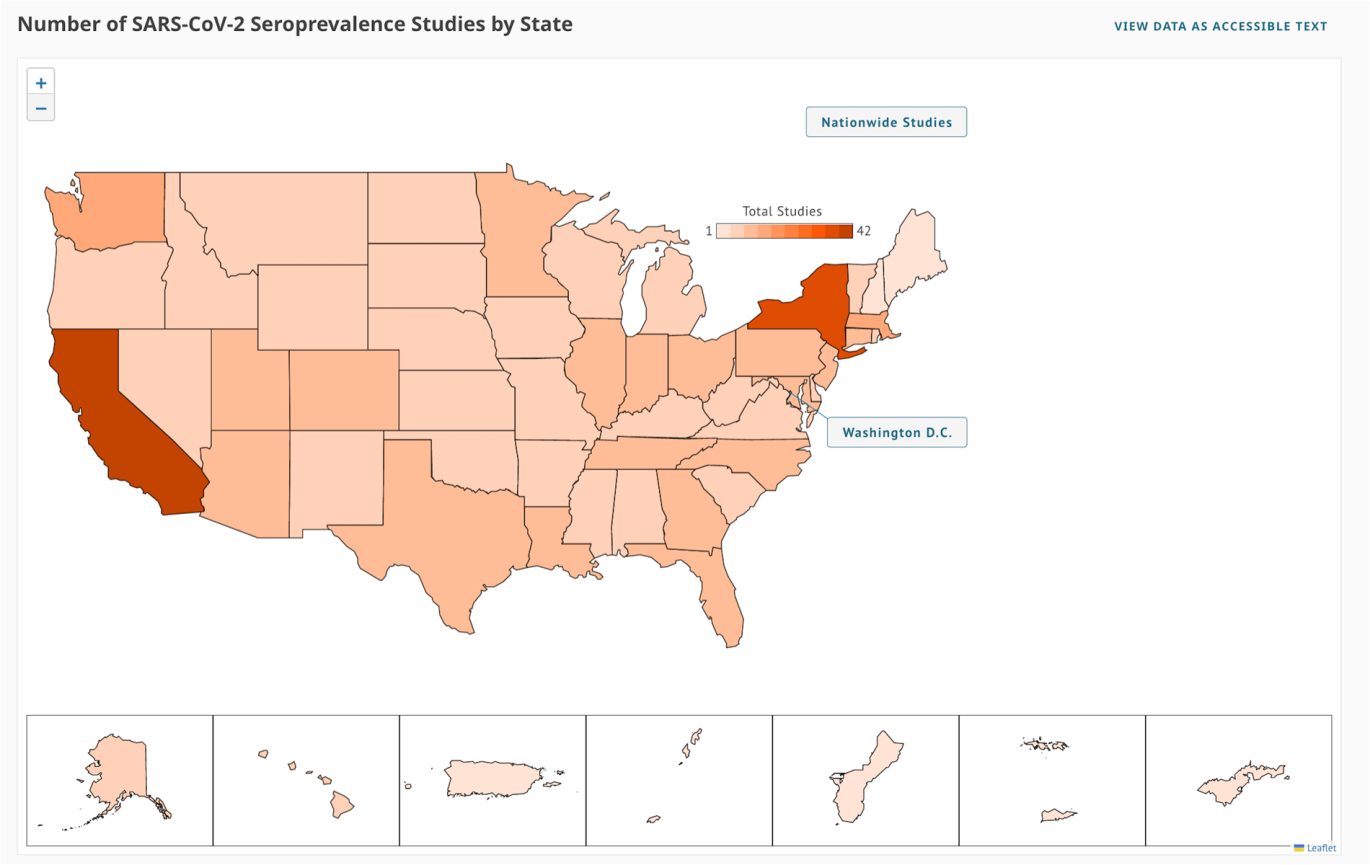
Screenshot of the Studies Map tool in COVID-19 SeroHub that allows users to view the geographic location of US seroprevalence studies.

**Fig. 4:**
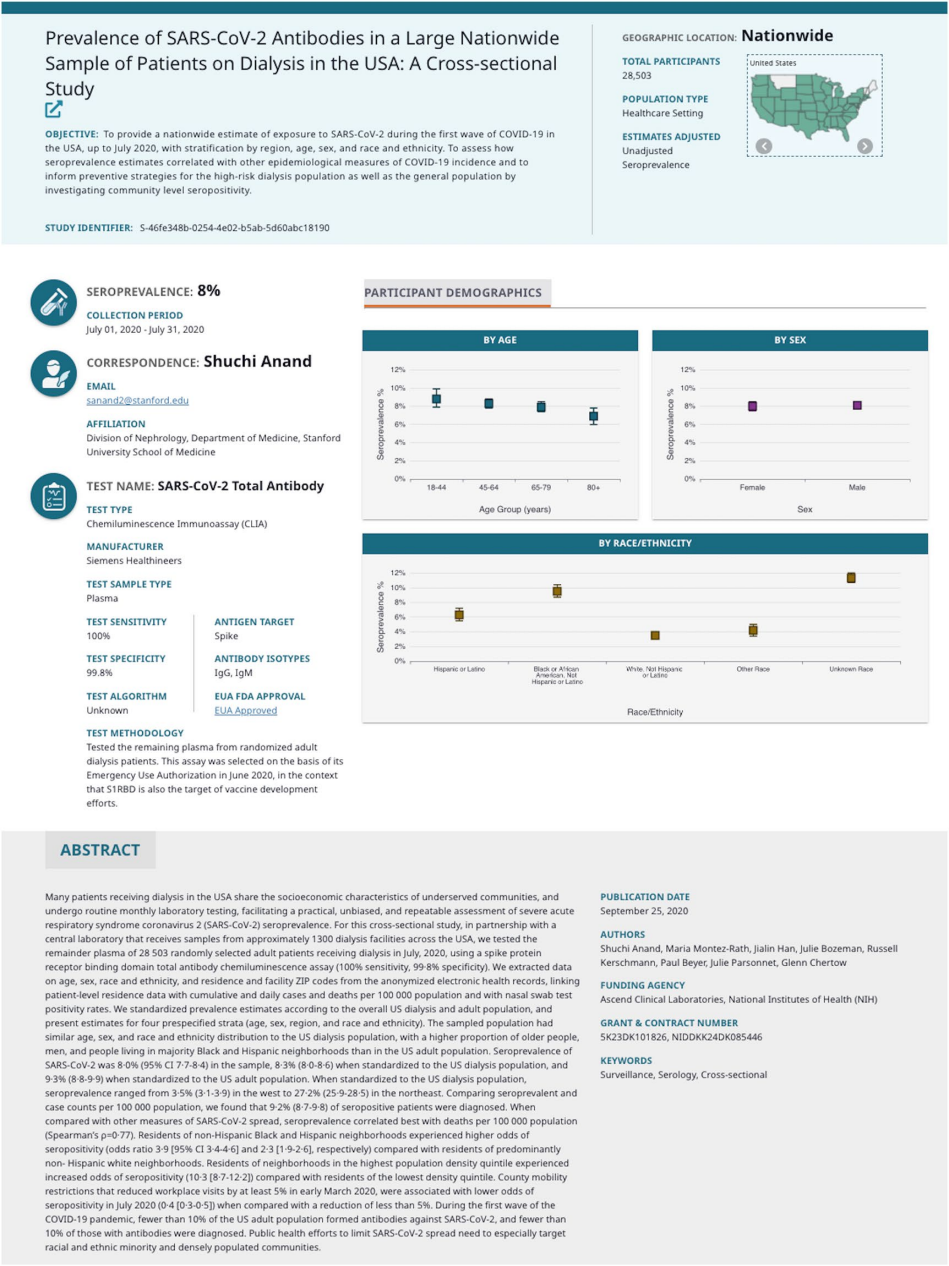
Screenshot of a sample individual study page in COVID-19 SeroHub.

To meet this need, the US National Cancer Institute (NCI), the US National Institute of Allergy and Infectious Diseases (NIAID) (both part of the National Institutes of Health), and the US Centers for Disease Control and Prevention (CDC) developed the COVID-19 Seroprevalence Studies Hub data repository, or COVID-19 SeroHub (https://covid19serohub.nih.gov/). The COVID-19 SeroHub team systematically identifies published and ongoing SARS-CoV-2 seroprevalence studies in the United States (Fig. 1) and extracts a standardized set of data elements from identified studies. Seroprevalence results from each extracted study are then presented as points on a scatter plot (Fig. 2) allowing users to visualize US seroprevalence estimates over time, geography, age, sampled population, serology test characteristics, age, sex, race/ethnicity, and other variables. Users can also find studies conducted in specific states on a map (Fig. 3), and each study has an individual page with more detailed information and a live reference link (Fig. 4). As of September 1, 2022, COVID-19 SeroHub includes 35,823 seroprevalence results from 178 extracted studies. Extracted data can also be downloaded as a Microsoft Excel spreadsheet via the Seroprevalence Data tab or can be assessed by API. COVID-19 SeroHub has a digital object identifier (DOI) of (10.17917/3pz5-5m44)^18^. To help users keep track of the data posted on SeroHub, data available for download is tagged with a version number (currently 3.1.0) and posting date.

COVID-19 SeroHub is intended to allow monitoring of the spread of COVID-19 across the country, inform future studies and public health decisions, and identify scientific gaps and disparities. Over the past year, SeroHub has been visited more than 1 million times and has helped inform governmental and policy discussions regarding the pandemic. Moving forward, we plan to continue to identify studies, extract their information, and update regularly as long as the COVID-19 pandemic continues.

## Methods

Studies are identified by a standardized protocol that includes weekly searches of Clinicaltrials.gov, MedRxiv, Pubcrawler, LITCOVID, PubMed, and Serotracker^16^ using terms described in Table 1 and direct identification by our group. Researchers can also provide information to SeroHub about their study using a submission template (https://covid19serohub.nih.gov/public/COVID-19_SeroHub_Submission_Template.xlsx) and submit by e-mail. If needed, we contact researchers to obtain missing information about their study.

**Table 1:** Resources and Search Terms to identify Seroprevalence Studies in the U.S.

Resource	Search Terms
Clinicaltrials.gov	Serology,
	Coronavirus,
	sars-cov-2
	Antibodies,
	COVID-19
	Seroprevalence
	SARS-CoV-2
JAMA Network	Serology,
	Coronavirus,
	sars-cov-2
	Antibodies,
	COVID-19
	Seroprevalence
	SARS-CoV-2
LITCOVID	Filter by Country to focus on United States, search for Serology and COVID-19
MedRxiv	Serology,
	Coronavirus,
	sars-cov-2
	Antibodies,
	COVID-19
	Seroprevalence
	SARS-CoV-2
Pubcrawler	Pubcrawler is a free alerting service that scans daily updates to the NCBI Medline (PubMed) and GenBank databases. We use the same search parameters as for PubMed (see below)
PubMed	
1	(((Immunoglobulins[Mesh] OR antibody OR anti-body OR antibodies OR anti-bodies OR Serologic Tests[Mesh] OR Immunoassay[Mesh] OR Serology[Mesh] OR serosurvey OR sero-survey)))
2	((coronavirus OR corona-virus OR covid 19 OR covid-19 OR covid19 OR covid 2019 OR covid-2019 OR covid 2019 OR severe acute respiratory syndrome coronavirus 2 OR SARS-CoV-2 OR sars cov-2 OR sars-cov 2 OR sar cov 2 OR wuhan virus))**
3	(2019/12[Date - Publication]: 3000[Date - Publication])
4	1 AND 2 AND 3 AND 4
Serotracker	Filter by Location–United States of America and filter out News and Media studies

Figure 1 provides a flow chart indicating how the more than 1500 studies were identified and then evaluated for inclusion in COVID-19 SeroHub.

Upon identification, studies are reviewed independently by at least two reviewers to determine if the study meets inclusion criteria: includes novel seroprevalence data; was conducted in the United States; describes the population tested; and describes the serology test(s), including test name(s), the antigen target(s) (*e.g*., spike or nucleocapsid proteins), test type, and antibody isotype(s) (*e.g*., IgG, IgM, panIg) detected. The options available for each of these fields are listed in the Data Records section of this manuscript. Exclusion criteria include outbreak investigations (e.g., single site, transmission chain), studies conducted on known or suspected SARS-CoV-2 infected populations, studies describing assay development or validation, or studies aiming to understand immune responses to coronavirus infection.

Studies meeting these inclusion criteria are extracted using a standardized data template with predefined fields (see a list of fields in the Data Records section below) by at least two extractors. Consensus is required for each field. Data elements were adapted and expanded from a prior effort developed by the NIAID Centers of Excellence for Influenza Research and Response (CEIRR) network^19^. At least two infectious disease epidemiologists conduct the second level of review. For studies that appeared suitable, but failed to include key information, such as serology test used, attempts are made to obtain this information from study investigators. Approved studies are then posted to COVID-19 SeroHub. Data from preprints are reviewed upon publication in peer-reviewed journals and subsequent data releases from extracted studies are also added when available.

Seroprevalence data in the COVID-19 SeroHub are aggregated and we do not collect individual level results. Most included seroprevalence data were extracted from study publications or preprints. However, staff are flexible and other mechanisms are possible. For example, seroprevalence data from studies with many collection periods and study locations, such as the CDC Blood Donor and Commercial Laboratory Seroprevalence Surveys^4–6^ (https://www.cdc.gov/coronavirus/2019-ncov/cases-updates/geographic-seroprevalence-surveys.html), were obtained directly from investigators or imported via API. To help users keep track of the data posted on SeroHub, data available for download is tagged with a version number (currently 3.1.0) and posting date.

### Data storage and integration

Once extracted, the COVID-19 SeroHub system programmatically validates extracted data against the data template by comparing the JSON schema expected values to the extracted values. Validated study and collection and report metadata are stored as structured JavaScript Object Notation (JSON) files on Amazon Web Services (AWS) S3 buckets. JSON files are the standard data exchange format for an Extract, Transform, and Load (ETL) pipeline built using AWS Lambda (Python). JSON data are structured for search and then indexed in Elasticsearch.

### Data visualization

Currently, data visualizations include an Interactive Seroprevalence scatter plot (Fig. 2), a Studies Map (Fig. 3), Individual Study Pages (Fig. 4), a Study Information Data Table, and a Seroprevalence Data Table that includes extracted seroprevalence results from each study. Source data for each visualization is keyword searchable and filterable; therefore, users can create custom searches for seroprevalence study data. For searches, the secure API queries Elasticsearch, a fuzzy full-text keyword search engine. This ETL process standardizes unstructured data for machine-readable access through APIs. For visualizations, the User Interface (UI) calls APIs to produce data visualizations and search results on COVID-19 SeroHub instantaneously.

The default view is the Interactive Seroprevalence Tool (Fig. 2), a scatterplot where calendar time is plotted on the x-axis and seroprevalence on the y-axis. The scatterplot contains all extracted seroprevalence results plotted by the midpoint of the collection period for each sample. Users can choose which results to display. Filters are dynamically deployed based upon available data. For example, users can decide to examine seroprevalence results in a particular state by calendar time, by age, by sex, by race/ethnicity, among specific populations (general population, healthcare setting, long-term care facilities, other occupations, pregnant people, military setting), by test characteristics such as spike (which detects positive results due to infection or vaccine), nucleocapsid (which detects positive results most likely due to infection), or by a combination of these factors. Available filters are described in Table 2.

**Table 2:** COVID-19 SeroHub *Seroprevalence Tool Filters*.

Filter Category	Filter Sub-Category
Age	Children 0–17 years
	Adults 18–49 years
	Adults 50–64 years
	Adults 65 + years
Population	General Population
	Healthcare Setting
	Long-term Care Facilities
	Other Occupations
	Pregnant People
	Military Setting
Geography	Each of the 50 USA States, US Territories, and Washington DC
	Nationwide estimates
Report Type	Preprint
	Peer-reviewed Publication
	Other
Special Collections	10 Site Study^5^
	Nationwide Commercial Laboratory Seroprevalence Survey^4^
	Nationwide Blood Donor Seroprevalence Survey^6^
	All Others
Test Type	Chemiluminescence Immunoassay (CLIA)
	Chemiluminescent Microparticle Immunoassay (CMIA)
	Electrochemiluminescence Immunoassay (eCLIA)
	Enzyme Linked Immunosorbent Assay (ELISA)
	Fluorescence Immunoassay (FIA)
	Fluorescent Microbead-based Immunoassay (FMIA)
	Lateral Flow Immunoassay (LFIA)
	Microsphere Immunoassay (MIA)
	Other
	Photonic Ring Immunoassay (PRI)
	Unknown
Antigen Target	Nucleocapsid
	Spike
	Spike and Nucleocapsid
	Not Specified
Antibody Isotype	IgG/IgM
	IgG (only)
	IgM (only)
	PanIg

Users can view studies by state (Studies Map tab; Fig. 3). This visualization is produced by calling RESTful APIs that query Elasticsearch and return results to the eCharts library used to create the map. Clicking on any geographic area results in Elasticsearch returning a list of the studies that were conducted within the chosen state or territory.

Study Information and Seroprevalence Data, the third and fourth tabs on the SeroHub main page, are data tables that enable browsing of study level information. Similar to the visualizations, these datasets may be filtered to refine queries. There is also a flexible keyword search bar that leverages full-text search to return the most relevant results on all text data. Fuzzy search matches on related, closely spelled search terms.

Users may download all data into Microsoft Excel (Excel) file format by navigating to the Seroprevalence Data Tab and clicking on the “Download Seroprevalence Data” button. Alternatively, all data may be accessed via API. To help users keep track of the data posted on SeroHub, data available for download is tagged with a version number (currently 3.1.0) and posting date.

Each study has a dedicated individual study page that provides more detailed information and a link to the study reference (Fig. 4). Each of these pages can be accessed by links from the scatter plot, the study map, or the study information tab.

Every new feature on the site is reviewed through user acceptance testing by our interagency working group, featuring members from NCI, NIAID, CDC, and study extractors. All features are reviewed on a developmental tier before being deployed on the production site. The readability of the site format has also been tested against 508 accessibility compliance checking tools Axe Plugin.

## Data Records

We developed a minimum set of data elements to extract from each study reflecting typically available data fields. As described above, these data are all available at the SeroHub data repository (10.17917/3pz5-5m44)^18^. In addition to using visualization tools available in the COVID-19 SeroHub data repository, all extracted study data can be downloaded by users of the site, without restriction, by navigating to the Seroprevalence Data Tab and clicking on the “Download Seroprevalence Data” button or obtained via API, facilitating analysis via other statistical software and webtools.

As part of our standardized protocol, the following fields are obtained from each included study and are available for download:

### Data elements name

Definition.

### Study and report title

Title of the manuscript or data release. If none, use study title.

### Lead author

Last, First name of lead author in manuscript or data release.

### Lead author email

Email address of lead author in manuscript or data release.

### Lead author affiliation

Affiliation of lead author. We use a curated list of institutions for consistency.

### Corresponding author

Name and email address of corresponding author if different than lead author.

### National clinical trial identifier

The National Clinical Trial (NCT) identifier for the study, if available.

### Study status

Status of the study. Choices are: Planning, Ongoing, and Complete.

### Funding agency

The agency that provided funding for the study.

### Grant or Contract #

If applicable.

### Study objectives

The objective of the study in one sentence.

### Primary study design

The type of study performed. Choices are: cross-sectional surveillance survey, serial surveillance survey, where repeated cross-sectional surveys of a target population are conducted, and longitudinal surveillance survey, where a single group of participants are repeatedly given serology tests over time.

### Study population

What was the study population? Choices include: General Population (children, adults, blood donors), Educational Setting (college students, teachers, K-12 students, university employees), Healthcare Setting (nurses, physicians, hospital patients), Other Occupations (meat packers, private sector and government employees, first responders), Detention Centers (prison inmates and personnel), Long-Term Care Facilities (nursing homes, rehabilitation facilities, long-term chronic care facilities, inpatient behavioral health facilities), Military Setting (Air Force base residents, Navy ship residents), Pregnant People, and Not Reported.

### Sampling methodology

A short description of how participants were enrolled into the study. For example:

*High-risk healthcare workers employed by Regional Hospital in City, State were invited to participate in the study. Study participants completed a questionnaire and provided a blood sample*.

*This study included all patients who had surgery at Regional Hospital in City, State between May 10, 2020 and July 28, 2020. Residual blood samples were used for serologic testing*.

*Within each of the counties in a State, we selected a random census tract using cluster sampling and recruited households. Then, we randomly invited a resident from each household to complete a survey and provide a blood sample*.

### Collection state

State, district, or territory where the sample was collected.

### Collection county

County where the sample was collected. Can also include Districts, Islands, Municipalities, Parishes, and Villages.

### Collection city

City, town, or borough where the sample was collected.

### Number of participants

The number of unique subjects enrolled in the study.

### Collection frequency

Number of times samples were collected from participants. Choices are: Once, Twice, Biweekly, Monthly, and Multiple.

### Collection period

The calendar months and year in which the study was actively collecting samples from enrolled subjects. If studies have multiple collection events, please include each collection event on a different row.

### Age

The age range of participants in the study.

### Gender/Sex

Female, Male, Nonbinary, Intersex, Transgender, Other, and Unknown.

### Race

The race of study participants, such as American Indian or Alaska Native, Asian, Black or African American, Hawaiian or Other Pacific Islander, White, Multiracial, International, Other, or Unknown.

### Ethnicity

The ethnicity of study participants, such as Hispanic or Latino, Not Hispanic or Latino, or Multiethnic.

### Test manufacturer and name

The company that manufactures the test. If multiple companies or tests, please provide each separately. If a lab developed a test, (LDT), please specify.

### Emergency use authorization (EUA) approval

Note if test received Food and Drug Administration (FDA) EUA approval. https://www.fda.gov/medical-devices/coronavirus-disease-2019-covid-19-emergency-use-authorizations-medical-devices/eua-authorized-serology-test-performance.

### Test type

List the type of test used. Choices are: Chemiluminescence Immunoassay (CLIA), Chemiluminescent Microparticle ImmunoAssay (CMIA), Electrochemiluminescence Immunoassay (eCLIA), Enzyme Linked Fluorescence Assay (ELFA), Enzyme Linked Immunosorbent Assay (ELISA), Fluorescence Immunoassay (FIA), Fluorescent microbead-based immunoassay (FMIA), Lateral Flow Immunoassay (LFIA), Luciferase Immunoprecipitation System (LIPS), Microsphere Immunoassay (MIA), Photonic Ring Immunoassay (PRI), Photometric Immunoassay (PIA), Unknown, and Other.

### Antigen target

The antigen target of the serology test used. Choices are:

Spike, Nucleocapsid, Spike and Nucleocapsid, Spike and Spike RBD,

Spike N-terminal domain (NTD), Spike Receptor-binding Domain (RBD), Spike trimeric ectodomain (Trimer), Spike S1 subunit, Spike S2 subunit, Spike S1 and S2 subunit, Not Specified, and Other.

### Antibody isotypes

The antibody isotype of the serology test used. Choices are IgG, IgM, IgA, [IgG and IgM], [IgA, IgG, or IgM] (PanIg), and Unknown.

### Specimen type

Type of samples used in study. Choices are: Whole blood, Dried blood, Plasma, Serum, and Other.

### Test sensitivity and 95% confidence interval

Test sensitivity for the serology test used in the study. We preferentially extract these data from the study; however, if not available, we obtain from the FDA EUA site, or if necessary, other sources.

### Test sensitivity source

How was this determined? (e.g., per manufacturer or per in-house validation).

### Test specificity and 95% confidence interval

Test specificity for the serology test used in the study. We preferentially extract these data from the study; however, if not available, we obtain from the FDA EUA site, or if necessary, other sources.

### Test specificity

How was this determined? (e.g., per manufacturer or per in-house validation).

### Testing algorithm

Describe if an algorithm was used to define seropositivity in the study. For example, how were data from multiple tests used to define seropositivity?

### Analysis strategy

Are seroprevalence estimates unadjusted or adjusted by population weighting, sensitivity/specificity, antibody waning, or other factors?

### Reported Seroprevalence, 95% confidence interval, and number of participants tested

The seroprevalence results reported in the study. We extract overall seroprevalence, seroprevalence by study location, seroprevalence by collection period, seroprevalence by age, seroprevalence by race and ethnicity, seroprevalence by sex, seroprevalence by serology test used, and the number of participants in each subgroup.

### Data released (Y/N)

Whether the data from the study have been released publicly, for example through a publication.

### Study reference

Include web link or published reference.

### Report type

Can include Preprint, Peer-reviewed publication, and Other (such as press-release or image).

### Publication date

For peer-reviewed publications, this is the publication date. Alternatively, it can be the data of posting on a preprint server or release of a data report, depending on the source of data.

### Comments

Text describing anything else of interest related to the submission.

### Study keywords

Keyword(s) are generated that summarize the study’s approach or objective.

## Technical Validation

As described in Methods, we have included several procedures to ensure that we are accurately importing study data into COVID-19 SeroHub. Before posting, extracted study information is reviewed by several members of the extraction team. Then, these data undergo a second-level review by at least two infectious disease epidemiologists to ensure consistency across studies. All structured data are computationally validated against a JSON standardized data template before incorporation into COVID-19 SeroHub. Once posted, results are periodically checked for accuracy and completeness by the entire team and automated tests. We also incorporate feedback from study authors about the extraction of their studies and comments from the public.

## Usage Notes

COVID-19 SeroHub is intended to be used by researchers, policymakers, state and local public health officials, and the public to monitor the spread of SARS-CoV-2 infections and COVID-19 vaccinations across the country, facilitate evidence-based decision making, inform future studies, and identify scientific gaps and disparities. Demands for seroprevalence data continue to increase as the database grows and the pandemic evolves. For example, COVID-19 seroprevalence data informed discussion by the Advisory Committee on Immunization Practices of vaccine recommendations for children aged 5–11 years (https://www.cdc.gov/vaccines/acip/meetings/slides-2021-11-2-3.html).

By extracting and presenting seroprevalence data in a standard format, COVID-19 SeroHub helps users to efficiently review numerous studies and identify gaps. COVID-19 SeroHub also helps users to monitor the spread of SARS-CoV-2 over time by population and geographic area, important for identifying the populations and regions most vulnerable to the virus, such as locations where vaccination rates are low. Users can search through extracted studies using keywords as well as obtain extracted data by download or API. COVID-19 SeroHub does not have any restrictions on who can use or download data from the site. However, all data in COVID-19 SeroHub are stored as aggregate datapoints, and thus there is no risk to accidental release of individual or private data.

A strength of COVID-19 SeroHub is that studies are identified by comprehensive literature searches and from a wide variety of sources, including peer-reviewed publications, pre-prints, and government reports. COVID-19 SeroHub is also updated weekly and includes an ever-expanding number of studies and seroprevalence estimates. A flexible architecture allows the inclusion of both single data releases and biweekly or monthly data releases from ongoing studies, such as the large CDC nationwide commercial laboratory^4^ and blood donor studies^6^. Tools are provided that allow users to easily find specific studies and populations of interest, such as diabetes patients^20^, pregnant people^21^, crew on a fishing boat^22^, first responders^23^, and health care providers^7^. Standard data extraction and presentation protocols allow users to compare data across many studies. Seroprevalence estimates are reported verbatim from study publications allowing re-use of the data without concern that results may have been transformed from the original publication or data release. Studies can be compared across key fields including study population, age, sampling methodology, test performance and characteristics, antigen target, and other fields. Visualization tools allow users to examine the spread of the virus in different populations and geographic areas by calendar time. COVID-19 SeroHub’s tools allow users to view seroprevalence results by whether participants were tested for spike or nucleocapsid antibodies. Because vaccinations in the United States are directed against the spike component of the SARS-CoV-2 virus, this allows the user to examine results restricted to infection-induced seropositivity or to view results that include vaccine-induced seropositivity.

COVID-19 SeroHub has several limitations. It is manually curated, and as such, there is a lag between study availability and inclusion in COVID-19 SeroHub. The availability of APIs for some studies have greatly facilitated quick incorporation of study data into SeroHub. More widespread use of these and other tools for data sharing would be useful in the future. Extracting a standard set of data elements facilitates comparisons across studies; however, these are often a subset of the data available in individual studies. Although we include abstracts on each individual study page and provide a powerful search function, users interested in particular topics or sub-populations would benefit by consulting the original publications. To facilitate such explorations, individual study pages include links to the associated publications. Additionally, studies included in COVID-19 SeroHub have a range of study designs and sample sizes. Individual studies use many different sampling strategies and serology tests, each with distinct performance characteristics. For example, tests vary in their sensitivity and specificity, as well as in their ability to detect antibody waning over time^24^. Due to the complexity of comparing across studies, variation in how users wish to use the data, and importance of identifying studies conducted in various populations and geographic areas, we decided not to formally evaluate study quality in COVID-19 SeroHub. Substantial differences between studies speak to the importance of SeroHub and its ability to help users to identify and compare studies with different strengths and weaknesses that have been conducted at the same time in the same geographic area.

Although there is an urgent public health need for timely seroprevalence data, there are substantial lags in data availability. As of September 1, 2022, SeroHub includes 13,300 (37.5%) seroprevalence estimates from studies conducted in 2020, 21,400 60.3%) from studies conducted in 2021, and just 800 (2.3%) seroprevalence estimates from studies conducted in 2022. Although 156 studies provided data for 2020, only 21 studies provided data for 2021, with most results from the large ongoing CDC Blood Donor and Commercial Laboratory Seroprevalence Surveys^4–6^. Only one study includes data from 2022^4^ reflecting the Omicron variant surge in the US, with the most recent data for May-June 2022^25^. Even with the emergence of pre-print servers^26^, most seroprevalence results are released months after sample collection. Sustained efforts to increase the timeliness of data release are thus needed. Other fields, such as genomics, have moved to models where sequence data are rapidly deposited into established databases and analyzed in real-time. We believe that this is an important goal for serology studies as well and note that the infrastructure developed for COVID-19 SeroHub could facilitate the rapid release of seroprevalence data. For example, studies could submit their information while in the field and then release their data systematically to the public via API. Although perhaps too late for SARS-Cov-2, such infrastructure efforts should be considered as part of the response to future pandemics.

Additionally, despite the known importance of representative surveillance in the United States, we have not identified any truly nationally representative seroprevalence studies. Only a few representative studies have been conducted at either a state-wide or county-wide level and these studies were conducted early in the pandemic in 2020^9,10,27–29^. Most studies have recruited volunteer participants or special populations such as blood donors or dialysis patients, raising concerns about the representativeness of conducted studies. A wealth of data indicates that the impact of SARS-CoV-2 varies substantially by key demographic factors such as age and race/ethnicity^30–32^. Yet, there is considerable variability in how individual studies have categorized these important demographic variables. This lack of standardization challenges efforts to harmonize and present these important data. Seroprevalence also likely varies by socio-economic status, yet unfortunately few studies collected information about education or income.

Two years into the pandemic, seroprevalence studies continue to provide important insights into the spread of SARS-CoV-2 through the population as well as vaccination. For this reason, we expect to incorporate studies and add functionality to COVID-19 SeroHub well into the future.

### Disclaimer

The findings and conclusions in this report are those of the authors and do not necessarily represent the official position of the Centers for Disease Control and Prevention or the National Institutes of Health. Use of trade names is for identification only and does not imply endorsement by the Centers for Disease Control and Prevention.

## Data Availability

COVID-19 SeroHub uses custom code to store manually extracted data without processing. As described above, these data are downloadable by users via spreadsheet or API. Tools for visualizing extracted data were produced using HTML 5.0 and ECMAScript/Javascript and tested with Chrome Browser 90.x, Safari 14.x, and Firefox 88.x. The Apache eCharts 5.1.1 library was used to produce the Studies Map, Interactive Seroprevalence Tool, and Individual Study Page data visualizations. API code was produced using the Python version 3.8 on AWS Lambda, AWS API Gateway, and Elasticsearch 7.9. All code and cloud resources are secured in Federal Information Security Modernization Act (FISMA) compliant environments.
